# Advances in Biosensing Technologies for Diagnosis of COVID-19

**DOI:** 10.3390/bios12100898

**Published:** 2022-10-20

**Authors:** Sulaiman Alsalameh, Khalid Alnajjar, Tariq Makhzoum, Noor Al Eman, Ismail Shakir, Tanveer Ahmad Mir, Khaled Alkattan, Raja Chinnappan, Ahmed Yaqinuddin

**Affiliations:** 1 College of Medicine, Alfaisal University, Riyadh 11533, Saudi Arabia; 2 Laboratory of Tissue/Organ Bioengineering and BioMEMS, Organ Transplant Centre of Excellence, Transplant Research and Innovation Department, King Faisal Specialist Hospital and Research Centre, Riyadh 11211, Saudi Arabia

**Keywords:** COVID-19 diagnosis, biosensors, SARS-CoV-2, RT-qPCR, COVID-19 biomarkers

## Abstract

The COVID-19 pandemic has severely impacted normal human life worldwide. Due to its rapid community spread and high mortality statistics, the development of prompt diagnostic tests for a massive number of samples is essential. Currently used traditional methods are often expensive, time-consuming, laboratory-based, and unable to handle a large number of specimens in resource-limited settings. Because of its high contagiousness, efficient identification of SARS-CoV-2 carriers is crucial. As the advantages of adopting biosensors for efficient diagnosis of COVID-19 increase, this narrative review summarizes the recent advances and the respective reasons to consider applying biosensors. Biosensors are the most sensitive, specific, rapid, user-friendly tools having the potential to deliver point-of-care diagnostics beyond traditional standards. This review provides a brief introduction to conventional methods used for COVID-19 diagnosis and summarizes their advantages and disadvantages. It also discusses the pathogenesis of COVID-19, potential diagnostic biomarkers, and rapid diagnosis using biosensor technology. The current advancements in biosensing technologies, from academic research to commercial achievements, have been emphasized in recent publications. We covered a wide range of topics, including biomarker detection, viral genomes, viral proteins, immune responses to infection, and other potential proinflammatory biomolecules. Major challenges and prospects for future application in point-of-care settings are also highlighted.

## 1. Introduction

Severe acute respiratory syndrome coronavirus 2 (SARS-CoV-2) was first identified in China, and has grown into a jeopardizing pandemic [1,2]. SARS-CoV-2 is an enveloped, positive-sense, single-stranded genomic RNA (26–32 kb) virus. The viral envelope is mainly composed of three proteins. The spike (S) glycoprotein, found on the surface, consists of S1 and S2, and holds the receptor-binding domain (RBD) for ACE2 (angiotensin-converting enzyme 2). The envelope (E) protein is responsible for viral assembly, release, and pathogenicity [3]. The membrane (M) protein organizes the assembly and shapes the envelope [4]. It aids in morphogenesis and budding and carries immunogenic properties [5].

SARS-CoV-2 is primarily transmitted in droplets. After entering the respiratory tract, S-protein’s RBD binds to ACE2 on the epithelium. ACE2 receptors are widely present in human tissues, especially in the respiratory and gastrointestinal tracts [6]. Cellular proteases cleave spike proteins from the viral envelope, likely to facilitate membrane fusion and internalization of the viral genome. Viral RNA and proteins are expressed within the cytoplasm, allowing virion synthesis and exocytosis. Eventually, the cellular stressors and the immune response result in the apoptosis of the host cell. This causes immune dysregulation, driving hypercytokinemia, mucus buildup, and airway hyperplasia [7], creating the classic clinical presentation of COVID-19—fever, dry cough, dyspnea, and fatigue. Moreover, SARS-CoV-2 may compromise other tissues such as the heart, kidneys, liver, eyes, and nervous system. SARS-CoV-2′s asymptomatic incubation period is 2–7 days, whereby infection may spread [8].

Most treatments for SARS-CoV-2 focus on curbing its progression, while others—such as the antiviral medications Remdesivir (Veklury), Paxlovid, and Molnupiravir—promote recovery of the milder symptoms. REGN-COV2 (Casirivimab and Imdevimab), a cocktail of two noncompeting IgG1 antibodies, also shows capacity to lower viral load and hospitalization [9]. Additionally, dexamethasone is strongly recommended by the National Institutes of Health (NIH) COVID-19 Treatment Guidelines Panel in hospitalized patients who require supplemental oxygen or mechanical ventilation. If corticosteroids cannot be used, baricitinib plus remdesivir may be used in nonintubated patients [9].

One of the cheapest and quickest ways to screen for COVID-19 is by clinical presentation, such as temperature measurement [10,11]. Measuring temperature is by no means specific, but its convenience has allowed for its use in public places to aid in the isolation of sick patients [11]. Imaging has also been used in the diagnosis of COVID-19. It predominantly shows bilateral, diffuse, peripheral abnormalities, ground glass opacities (GGOs), and consolidation in CT and X-ray. These signs appear to play a determinant role in patient prognosis and may signal a more severe disease [12,13,14,15]. It is also useful in screening patients and for follow-up after recovery [12]. However, it has low specificity, shown by the relatively indistinguishable nature of COVID-19 images compared to SARS-CoV and MERS [12]. Alternatively, serum analyses can also aid in diagnosis. Patients commonly present with lymphopenia, leukocytosis, elevated CRP, and signs of coagulopathies, though enzymes such as LADH, ALT, and AST can also be elevated [16,17,18]. Although these markers are nonspecific to inflammatory diseases, they can aid in the management and prognosis of patients [16,17].

This review aims to shed light on updates regarding the development of biosensors in the diagnosis of COVID-19, and how they offer potential as a cheaper, faster, and more convenient diagnostic tool compared to conventional methods.

## 2. Conventional Methods for Detecting SARS-CoV-2

**Figure 1:**
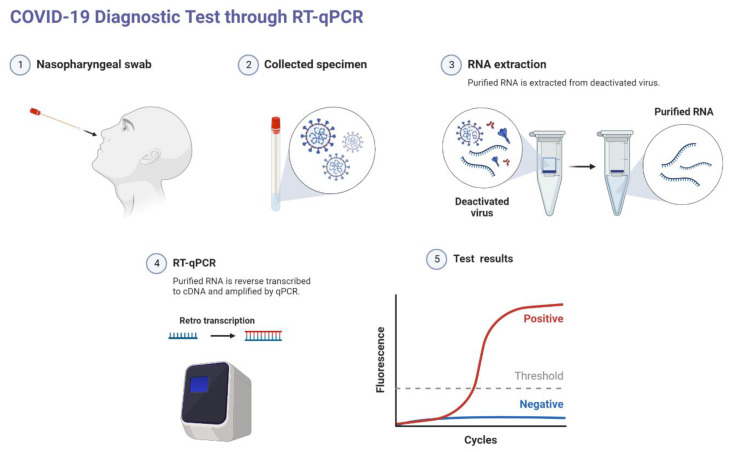
SARS-CoV-2 diagnosis by nucleic acid amplification method, qRT-PCR. (**1**,**2**). Nasopharyngeal swabs were taken from the patients. (**3**). RNA extracted/purified by RNA purification kit. (**4**). Extracted RNA is reverse transcribed to complementary DNA and amplification using the target-specific primers. (**5**). Quantitative real-time PCR (qRT-PCR) to identify the positive samples. Conducted to identify positive samples in real-time from the fluorescence of the dsDNA intercalating dyes such as SYBR green.

The most specific means for the diagnosis of SARS-CoV-2 infection is through biomarker detection [10]. Currently, the gold standard diagnostic method for SARS-CoV-2 is using enzyme-mediated amplification of specific genomic materials, such as DNA and RNA. The aim of Reverse Transcription Polymerase Chain Reaction (RT-PCR) is to detect the viral RNA, in which the RNA is reverse transcribed to complementary DNA (cDNA); then, the cDNA is amplified and, in qRT-PCR, quantified, as depicted in Figure 1. Commercially available COVID-19 PCR kits target the conserved regions of RdRp, E, N, or ORF1 genomic sequences. PCR tests are a highly sensitive, specific, and reliable method for clinical application. This method can detect as low as 0.689 copies/µL [19]. However, it takes a long time to obtain the results (one to six hours) in addition to the time taken to transport samples to the specialized laboratories and delays in the screening, which often occur due to the massive population of the samples. It is also very costly, requires trained personnel, and comes with a risk of false negatives [20]. However, the risk of false negatives seems to be affected by time because of exposure, collection technique, as well as the source of the sample, where lower respiratory tract samples show higher sensitivity compared to upper respiratory tract samples [10,21]. As such, clinical presentation, along with laboratory and chest imaging, may be used for the diagnosis of inpatients with a high clinical index of suspicion.

Lateral Flow Immunoassays (LFIA) and ELISA have been also used to detect IgM and IgG antibodies in patient serum, which may be detected even after the infection subsides. However, SARS-CoV-2 diagnosis from these methods is still challenging due to its complex mechanisms and low sensitivity and specificity, particularly in samples with low viral load. Additionally, patients typically take 7 to 11 days to seroconvert after exposure, preventing the detection of a current infection [22]. Alternatively, the detection of protein has recently been conducted through rapid antigen tests (RATs) by use of ELISA or LFA techniques to detect viral proteins from patient serum [2]. In spite of the low sensitivity of these methods compared to RT-PCR [18,23,24], their lower cost and ease of use make them appealing for rapid screening and in places of limited laboratory facilities.

Ultimately, there is a need for novel methods for the point of care application. The proposed techniques must easily detect the virus with limited resources. Biosensors can overcome the limitation of the current method of diagnosis, and they are straightforward and convenient to detect the target molecules from COVID-19-infected patient samples.

## 3. Principles and Application of Biosensors

**Figure 2:**
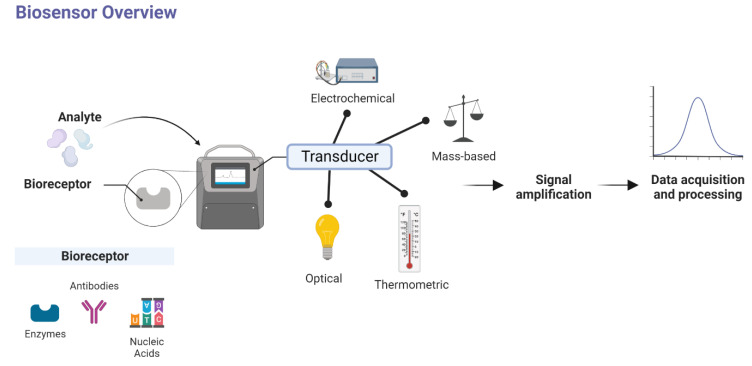
Schematic diagram and operational principle of biosensors. The sample is introduced to the biosensor; the bioreceptor interacts with the target molecule. The transducer coupled with the receptor transfer the respective signal to the amplifier, then the amplified signal is converted into readable signals.

Biosensors are reliable, sensitive, specific, rapid, user-friendly, low-cost in production, and can be used at the point of care by anyone without the need for intense training [25]. Biosensors are portable analytical devices containing biological recognition molecules (antibodies, enzyme aptamers, or nucleic acids) that are integrated with the transducer and the detector, giving a signal when the target analyte is recognized by the biosensor device. All the possible targets of SARS-CoV-2 are shown in Figure 2 for fabricating the different kinds of biosensors based on the target analytes. When the SARS-CoV-2 virus binds to the angiotensin-converting enzyme-2 (ACE-2) receptor, an immune response is immediately mediated by IgG and IgM antibodies. These antibodies can be used as clinical biomarkers for the diagnosis of COVID-19 as an alternative to genomic sequences. They can also be used for plasma therapy [26,27]. Antibody-based biosensors (immunosensors), nucleic acid-based biosensors (Genosensors), and whole cell-based biosensors are common sensors used for the diagnosis of viral particles [28,29]. The recognition receptor selectively binds to the specific target from the virus with high affinity, which can be an antibody raised against certain viral proteins, aptamers selected against the viral proteins, the whole viral particle, viral antigens, or the complementary DNA against the specific genomic sequence of the virus. When the bioreceptor interacts with the target analytes, the transducer converts a series of changes (optical, electrochemical, thermal, mass, or field effect transistor) into a measurable signal. Finally, the signal amplifier converts the measured signal into a readable signal, which can quantify the target present in the sample. However, not all the biosensors are integrated with signal amplifier and signal reader, such as lateral flow assay. 

## 4. Biomarkers in COVID-19

**Figure 3:**
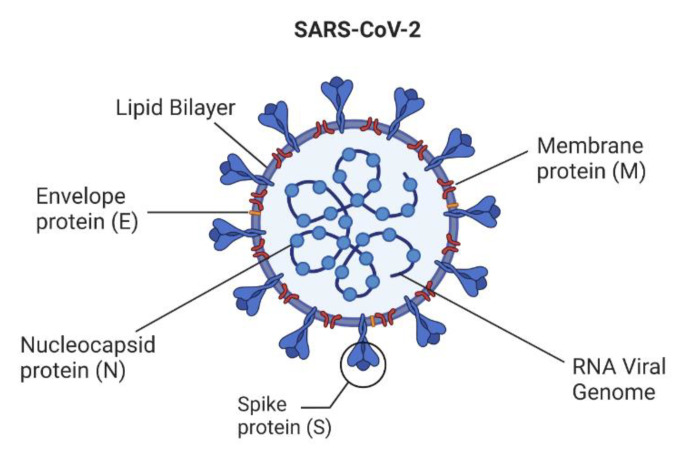
Schematic representation of various genomic parts and their organization in the SARS-CoV-2 viral particle.

One of the most common methods of diagnosing COVID-19 is through the detection of biomarkers. The three primary biomarkers detected in such strategies are proteins, nucleic acids, and antibodies. Proteins are excellent targets for COVID-19 detection in patients. For example, the spike protein’s antigenicity is exploited in multiple immunoassay kits for the detection of IgG and IgM antibodies [2]. The N-protein has been used for RATs to detect SARS-CoV-2 in patient samples [18]. Figure 3 represents the various possible biomarkers and other genomic components organized in the SARS-CoV-2 viral particle. Viral nucleic acid is also a potential biomarker, detected through nucleic acid replication methods, primarily PCR. It is a sensitive biomarker in the early stage of infections and/or high viral load, but can present with false negatives in the postinfection stage [10,20,21].

### 4.1. Nucleic Acid-Based Biosensors

**Figure 4:**
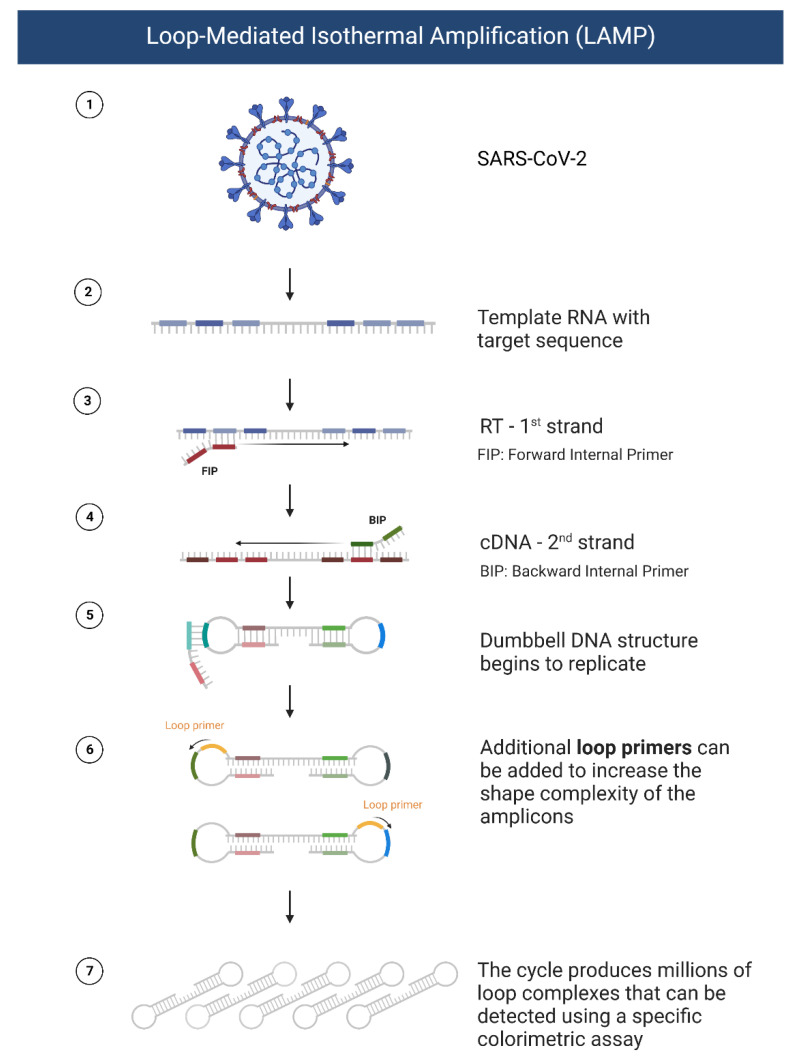
Schematic representation of loop-mediated isothermal amplification of SARS-CoV-2 RNA amplification.

There are several molecular methods—polymerase chain reaction (PCR), quantitative real-time PCR (qRT-PCR), clustered regularly interspaced short palindromic repeats (CRISPR), reverse transcription loop-mediated isothermal amplification (RT-LAMP), next-generation sequencing (NGS)—used for the diagnosis of COVID-19. The standard nucleic acid amplification methods involve RNA extraction from specimen collection by nasal swab, lysis, purification, amplification, and detection. It is a cumbersome multistep process needing different reagents for each step [30]. Loop-mediated isothermal amplification (LAMP) is a widely recognized amplification method that was developed by Notomo et al. in 2000 [31]. LAMP works by amplification of samples at a fixed temperature through cycles of two types of elongation reactions [32]. A stepwise representation of LAMP processes is seen in Figure 4. LAMP requires only a single enzyme to be used at a constant temperature throughout the reaction, reducing the cost of this method and possibly allowing the development of a portable detection method. Four to six primers are used, and they bind to specific gene regions; this gives LAMP its high specificity compared to other detection methods. LAMP sensitivity is similar to nested PCR. Moreover, the process of amplification takes about one hour to complete, which can be reduced to up to one-third of the original LAMP reaction by the design and use of specific loop primers [33]. Reverse transcription to derive cDNA is possible on SARS-CoV-2 since the nucleic acid in the virus is RNA. Colorimetric visualization of SARS-CoV-2 using RT-LAMP is possible with the addition of DNA intercalating object SYBR green dye [34]. In a systematic review by Pu et al., RT-LAMP showed a sensitivity and specificity of 92% and 99%, respectively, compared to the 96% sensitivity and 100% specificity of RT-PCR [35]. Furthermore, the dual color RT-Lamp technique has been developed for detecting the COVID-19 N gene in RNA samples, isolated from 768 pharyngeal swab specimens, with a sensitivity and specificity of 97.5% and 99.7%, respectively [36]. Similarly, RT-LAMP-based colorimetric assay was developed with sensitivity and specificity comparable to qRT-PCR. The LAMP primers target the RpRd gene amplification. The method is validated in 2120 clinical samples. The specificity and the sensitivity is 95.74% and 99.7%, respectively [37]. 

Newly developed methods are compared to RT-PCR in their specificity and sensitivity. Other methods can have an edge over PCR-based methods in terms of cost and time of analysis [34]. For example, a multiplex point of care RT-PCR can provide the results by inserting the nasopharyngeal or oropharyngeal swab directly into the device without a laboratory setting and well-trained technicians. Seven different target genes of SARS-CoV-2 (rdrp1, rdrp2, E-gene, n-gene, n1, n2, and n3) are analyzed at a sensitivity and specificity of 94% and 100%, respectively. This method, named Covidnudge, is widely used in UK hospitals [38]. Moitra et al. described an alternative colorimetric method based on the gold nanoparticle (AuNPs) optical properties and utilized it for the specific detection of the SARS-CoV-2 N-gene (nucleocapsid phosphoprotein) within 10 min. The selectivity of this method was tested in the presence of MERS-CoV viral RNA with a LOD of 0.18 ng/L. Selective, visual naked-eye detection of the COVID-19 virus without any sophisticated instruments has been achieved [39]. Another paper described the development of a highly sensitive one-step droplet digital RT-PCR (RT-ddPCR) multiplex assay for the simultaneous detection of different genes (N, E, and RdRp) of SARS-CoV-2. Patient-derived mRNA of the housekeeping gene was used for assay quality control. RT-ddPCR is superior to the gold standard RT-PCR in the clinical setting due to its high sensitivity, ease of use, and high throughput multiple target screening [40]. In addition, specific detection of SARS-CoV-2 RNA by nucleic acid amplification technology has been developed for POCT using fluorescence signals. Fluorescently labeled molecular beacons are used to identify the specific region [30]. 

Another isothermal amplification method is recombinase polymerase amplification (RPA), which represents a hugely versatile single tube isothermal alternative to PCR for the development of fast and portable nucleic acid detection assays. The amplification is carried out at a constant temperature; hence, there is no need for expensive thermal cyclers for heating and reheating [41]. RPA results are much faster than many diagnostic methods, with results being generated in as little as 3 min [42]. Once initiated, the amplification reaction progresses rapidly, so that just beginning with a few target copies of DNA, the highly specific DNA amplification achieves detectable levels within minutes [41]. RPA is highly specific, with 100% specificity for the target sequence in most instances. However, RPA has been described to be dependent on both the quantity and distribution of mismatches in the sequence of closely related DNA molecules. Hence, it is not possible to differentiate one or more mismatches varying based on their distribution [43]. RPA’s tolerance to mismatches, despite limiting its usefulness in sequence-specific primers, can be utilized to improve techniques that detect the presence of evolving variant pathogens when distinguishing from the wild-type target is not needed [44]. Lu et al. demonstrated the diagnosis of SARS-CoV-2 virus by microfluidic-integrated lateral flow RPA (MI-IF-RPA) successfully within 30 min. The RPA reagents are mixed in the buffer and the reaction was performed at 42 °C for 15 min in a thermoblock. After the reaction, the amplified products were subjected to a lateral flow assay strip conjugated with antibodies for visual detection. The LOD of this method is 1 copy/µL, with the sensitivity and specificity of 97% and 100%, respectively [45]. In another set of studies, the microfluidic chamber is integrated with LAMP and RPA for the detection of SARS-CoV-2 and measles virus (MV) in parallel simultaneously by the fluorescence signal change. This point-of-care testing method showed 100% clinical specificity and sensitivity for MV and 94.12% specificity and 95.83% sensitivity for SARS-CoV-2 with the LOD of ten copies within one hour [46]. 

RPA process employs three core enzymes, a recombinase, a single-stranded DNA-binding protein (SSB), and a strand-displacing polymerase. Recombinases are capable of pairing oligonucleotide primers with homologous sequences in duplex DNA. An SSB binds to the displaced DNA strand and stabilizes the resulting D-loop, preventing the primers from being displaced. Finally, the strand displacing DNA polymerase begins DNA synthesis, where the primer is bound to the target DNA.

Recombinase-aided amplification (RAA) is very similar to RPA; it utilizes recombinase, SSB, and DNA polymerase under isothermal conditions at 37 °C to form a polymer with primers. Double-stranded DNA unwinds at a sequence homologous to the primer. Strand replacement occurs between the primer and template with the action of SSB and DNA polymerase, and amplification of new DNA fragments occurs rapidly in vitro. This reaction occurs in the presence of SSB and condensation agent polyethylene glycol, and this process is continuously repeated to achieve the final efficient nucleic acid amplification. The target gene can be amplified to a detectable level within 5–10 min [47,48]. Zheng et al. developed an RAA-based method for the detection of COVID-19 which targets the nucleocapsid (N) gene of SARS-CoV-2. They designed specific primers and probes for reverse transcription recombinase-aided amplification coupled with lateral flow dipstick (RT-RAA/LFD). The point-of-care test assay that was designed offered 100% specificity and sensitivity in the detection of clinical samples compared to RT-qPCR [49]. Real-time reverse transcription RAA (RT-RAA)-based POCT was developed for the detection of SARS-CoV-2. The primers are designed to target the highly conserved region, 172 bp in the orf1ab gene of SARS-CoV-2, which covers 98% of SARS-CoV-2 strains. This method is tested for SARS-CoV-2 and eight other respiratory RNA viruses, and it showed high specificity and sensitivity to SARS-CoV-2 compared to other viruses. It can detect as low as 0.00048 copies/µL [50]. 

Pulse-controlled amplification (PCA) is a genome-based method following the same general principle as PCR but it is up to ten times faster [51]. PCA does not require RNA extraction, and the device used for carrying out PCA is lightweight and portable, making it a suitable POCT for the detection of COVID-19. Zwirgelmaier et al. showed that simultaneous detection of eight swab samples in under 25 min and with a 100% sensitivity was performed using a prototype PCA. The sensitivity of this assay is 100%, and it can detect the viral load of 1600 copies/µL [52]. 

Clustered regularly interspaced short palindromic repeats (CRISPR) perform gene-targeting and amplification using enzymatically disabled CRISPR-associated protein 9 (Cas9), associated with a specific single-guide RNA and immobilized on the transistor. This results in a label-free nucleic acid testing device with an easily measurable output [53]. Gootenberg et al. and Li et al. developed the SHERLOCK [54] and HOLMES [55] methodologies, respectively, that allowed for the detection of sequence-specific nucleic acids via collateral cleavage of the single-stranded nucleic acid probe after amplification [56]. These methods are programmable, allowing them to be modified for the detection of various targets [57]. CRISPR-Cas technology can detect specific gene sequences of COVID-19 within one hour. Furthermore, the LOD is between 10 and 100 copies/µL [58,59]. CRISPR/Cas9-mediated triple-line lateral flow assay (TL-LFA) integrated with RT-RPA for rapid and simultaneous detection of two different gens, envelope (E) and open reading frame 1ab (Orf1ab), in a single strip test. This assay showed a sensitivity of 100 copies per reaction (25 µL) for the genes obtained from cell-cultured SARS-CoV-2 and SARS-CoV-2 viral RNA standards. A total of 64 nasopharyngeal swab samples analyzed by CRISPR/Cas9 TL-LFA showed 100% negative predictive agreement and 97.14% positive predictive agreement [60].

In a wide range of applications, localized surface plasmon resonance (LSPR) has become a potent optical detection tool for probing label-free biomolecular interaction in real-time. SPR data provide a straightforward, accurate, and label-free method for quantitative analysis [61]. Alternatively, a newer method for utilizing light as the energy source that drives chemical reactions, photo-thermal catalysis, can increase reaction rates and alter selectivity patterns using the synergistic combination of photo- and thermo-chemical contributions of sunlight, even in mild operating circumstances. This photothermal effect is utilized in the detection of COVID-19, as shown by Qui et al. Briefly, nucleic acid hybridization can facilitate sensitive recognition of specific sequences from SARS-CoV-2 using two-dimensional gold nanoislands (AuNIs) and complementary DNA receptors. This arrangement of nanoabsorbers allows the plasmonic chip to transduce signals via in situ hybridization and generate local plasmonic photothermal (PTT) heat for highly accurate and sensitive detection of SARS-CoV-2 [62]. 

Electrochemical biosensors are another extremely promising form of biosensors for highly accurate point-of-care SARS-CoV-2 detection. It is common practice to employ electrochemical biosensors to identify viruses, proteins, tiny molecule antibodies, and nucleic acids [63]. The target is recognized in electrochemical sensors (EISs) through either an antigen-antibody reaction, DNA, RNA, peptide nucleic acid (PNAs) hybridization, or aptamers-based binding, each of which has great selectivity and sensitivity for the detection target [64]. Label-free EISs have drawn the most interest amongst them because of their extreme sensitivity, capacity for quick electrochemical sensing, and characterization of different analytes, including virus antigens, antibodies, and RNA [65]. Kashefi-Kheyrabadi et al. have developed nucleic acid amplification-free multiplex electrochemical biosensors for the rapid detection of SARS-CoV-2. They used a four-way junction, universal DNA hairpin probe duplexed with the two adopter strands and target RNA. One of the adopter strands is coupled with the redox mediator (methylene blue or ferrocene) for electrochemical signal sensing. This sensor can detect S and orf1ab genes simultaneously with the LOD of 5.0 and 6.8 ag/µL, respectively, within one hour. This is highly specific and can differentiate closely associated RNA with a single nucleotide addition in the sequence. The multiplexed sensor can be used in the point of care for real-time testing to diagnose COVID-19 [66].

Zhao et al. developed a calixarene functionalized graphene oxide electrochemical sensor for sensitive detection of SARS-CoV-2 RNA without amplification or reverse transcription. Super sandwich-type recognition technology was applied for recognizing the SARS-CoV-2 RNA, with a LOD of 200 copies/mL [67]. This device can detect as low as 26 fM, with high specificity which can distinguish the single base mismatch in the RNA sequence [68]. Heo et al. have designed an electrochemical biosensor for the detection of SARS-CoV-2 RNA via CRISPR/Cas13a transcleavage reaction. This device can detect ORF and S gene with the LOD of 4.4 × 10^−2^ fg/mL and 8.1 × 10^−2^ fg/mL, respectively. This is method is validated with RNA spiked in artificial saliva with 96.54–101.2% agreement [69]. Lab-on-a-chip for the simultaneous electrochemical detection of SARS-CoV-2 RNA and antibodies in saliva and plasma have been achieved within two hours. The three-dimensional printer device automatically extracts, concentrates, and amplifies SARS-CoV-2 RNA from the saliva directly. This device is integrated with Cas12a-based enzymatic detection of SARS-CoV-2 RNA via LAMP and ELISA on the electrode conjugated with RBD of spike S1 protein. This portable, low-cost multiplex electrochemical sensor would be used at the point of care applications for the diagnosis of COVID-19 [70]. The characteristics of various NAAT used to diagnose COVID-19 are summarized in Table 1.

### 4.2. Biosensors for the Detection of Antigens (Proteins)

Immunoassays are a convenient method for detecting the SARS-CoV-2 virus in the infectious stage [84]. Since immunoassays can diagnose the antigens in less than an hour and have detection ranges between fg/mL to μg/mL, they enable effective measurements of viral proteins such as S-glycoproteins, M-glycoproteins, E-proteins, and N phosphoproteins. N-protein is utilized as an early signal since it permits SARS-CoV-2 to be identified up to one day before symptoms appear [84]. Generally, to identify a biological molecule (SARS-CoV-2), the target molecule (viral protein) binds to the biosensor. The transducer with embedded nanostructures then turns the detection into an electrical signal identifiable by the detector [30]. Since the target protein in affinity sensors is detected on the device’s surface, the creation of such sensors requires the development of a surface with the right protein recognition capabilities, such as molecularly imprinted polymers (MIPs) [85]. In one study, the sensor was a disposable MIP-modified thin-film electrode to detect N-protein with a limit of detection and quantification of 15 fM and 50 fM, respectively [74]. They also have the benefit of being less expensive and more stable, and they can be built on protein-imprinted polymers such as polypyrrole and other electrochemically deposited polymers. Several signal detection techniques can be applied. MIPs can be built for small and low molecular weight compounds, making them appropriate for sensor design. Molecularly imprinted polyphenylenediamine-based electrochemical sensors for the detection of SARS-CoV-2 proteins, specifically the N-protein, have recently been developed using this technology, demonstrating the effectiveness of MIPs for the detection of some virus proteins. It should be emphasized that MIP-based sensors can detect even short DNA-based oligomers, making MIP-based sensors appealing for DNA and possibly RNA fragment determination [74,85]. 

The S-protein can be detected through its functional S1 subunit by a biosensor based on the bioelectric recognition assay (BERA). This S1 subunit interacts with ACE-2 in host cells. Bioelectric property changes by the interaction of the S1 functional subunit with the antibody. This method offers a quick reaction against the SARS-CoV-2 nucleocapsid protein with LOD of 1 fg/mL, and no cross-reactivity was noticed [71]. One method for identifying S- or N-proteins relies on the utilization of secondary antibodies along with alkaline phosphatase and magnetic beads as immunological labels and immunological chain support, respectively, resulting in LOD of 19 ng/mL and 8 ng/mL in untreated saliva respectively, for S- and N-proteins. The analytical features of the electrochemical immunological test were investigated for S- and N-proteins in the buffer and untreated saliva [72]. A sensitive graphene field effect transistor (GrFET) is paired with a highly selective antibody–antigen interaction to build a coronavirus immunosensor that allows simple and rapid screening/diagnosis of new coronaviruses. These GrFET immunosensors can promptly (~2 min) identify and effectively capture the SARS-CoV-2 S1 protein in a real-time, label-free modality with LOD down to 0.2 pM [73]. This sensor was created by using a 1-pyrenebutyric acid N-hydroxysuccinimide ester to couple an antibody against SARS-CoV-2 S-protein to graphene from an FET. This method was used to detect the S-glycoprotein. SARS-CoV-2 was identified by the FET system based on changes in the channel surface potential and the consequential effect on electrical response. In order to detect the targets, the gate surface of FETs is covered with a layer that may be changed using biomolecules [86]. To detect SARS-CoV-2 spike protein S1, graphene FET was coated with an antibody of SARS-CoV-2 spike S1 subunit protein (CSAb) or (ACE2). Partially positively charged S1 protein binding to the CSAb/ACE2 receptors on the graphene surface alters the conductance/resistance of the GrFET sensing platform. Because of its greater affinity, CSAb-modified GrFET demonstrated improved sensitivity [86,87]. 

**Figure 5:**
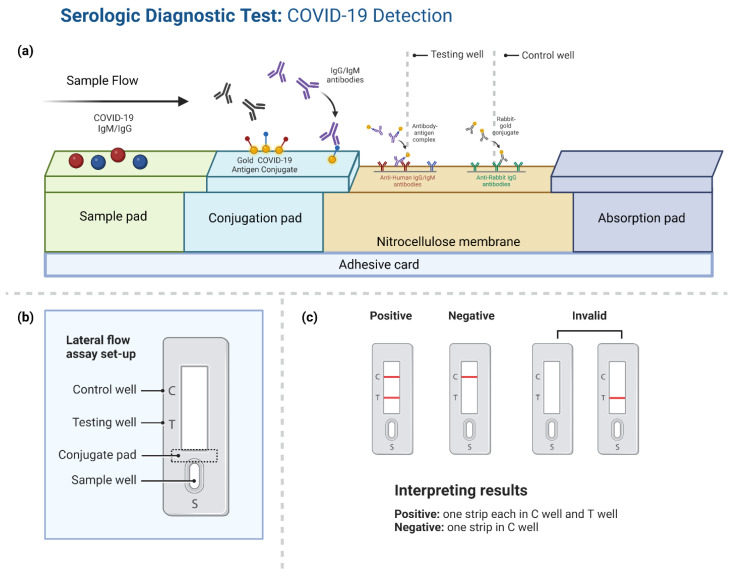
(**a**) Pictorial representation of lateral flow immunoassay working principle. (**b**) The assay setup in the commercial test pad and (**c**) Interpretation of assay results positive/negative and the stability of the testing pad.

LFIA and chemiluminescence immunoassay (CLIA) are two typical antigen detection technologies. The LFIA method uses colloidal gold test paper to identify antigens or antibodies; it is accessible, quick, and easy to read, but is only effective for qualitative detection and lacks sensitivity. The working principle of LFIA is represented in Figure 5. ELISA and CLIA are both quantitative procedures that use either enzymatic or chemical luminous agents to identify antibodies or antigens. Mekonnen et al. found that CLIA, ELISA, and LFIA have a sensitivity/specificity of 92/99%, 86/99%, and 78/98%, respectively [88]. Optical techniques have also been utilized to identify SARS-CoV-2 proteins, in which antibodies anchored to AuNPs, enzymes, or colored nanobeads are used. Their reaction with viral proteins results in changes in absorbance or the detection of a color signal in the visible spectrum. A complex of the N-protein, the anti-SARS-CoV-2-N antibodies, white microbeads, a second antibody, and a red nanobead enter the observational zone; the red color indicates the presence of the antigen (positive), while the white color indicates the negative. This device has LOD of <100 copies/mL in nasal samples [75]. Additionally, SARS-CoV-2 protein was recently detected using mass spectroscopy. Before experimenting, the protein was extracted and digested. SARS-CoV-2 protein was then estimated and determined while the protein was being fragmented. Nevertheless, this technique has certain drawbacks: it is time-consuming, requires an expensive apparatus and skilled workers, and takes about three hours to complete the detection procedure [89]. Sao et al. developed a field effect transistor-based biosensor (FET) for the detection of SARS-CoV-2 Spike protein. An FET biosensor is coupled with a spike protein-specific antibody on the graphene sheet. SARS-CoV-2 determination from the sample is used directly without any preprocessing. This method is more specific, as there is no cross-reactivity with the MERS-CoV antigen. The FET biosensor could detect the S-protein as low as 1 fg/mL in PBS buffer and 100 fg/mL in the clinical transport media [90]. An electrochemical immunosensor for the detection of SARS-CoV-2 N-protein in nasopharyngeal samples was developed using a label-free square wave voltammetry-based biosensing platform. SARS-CoV-2 nucleocapsid protein (N-protein)-specific antibody was conjugated on the screen-printed carbon electrodes coated with gold nanoparticles. The change in the electrochemical signal of the immunosensor upon binding the N-protein with the immobilized antibody is used for the determination of the N-protein present in the sample. The sensor could detect SARS-CoV-2 N-protein as low as 0.4 pg/mL. SARS-CoV was cross-reacted with the SARS-CoV-2 biosensor; however, there is no significant interaction with HCoV, MERS-CoV, Flu A, and Flu B. The sensor was successfully validated with clinical samples; the results were comparable with the RT-PCR results [76]. Rapid detection of SARS-CoV-2 spike 1 (S1) protein using ACE2 receptor-matched pair with commercially available antibodies by LFIA. This method is highly specific and there is no cross-reactivity with SARS-CoV S1 or MERS-CoV S1 protein with the LOD of 1.86 × 10^5^ copies/mL of COVID-19 patient clinical specimens [91]. Shao et al. have developed a SARS-CoV-2 antigen (Ag) FET nanobiosensor using semiconducting (sc) single-walled carbon nanotube (SWCNT) conjugated with antibodies specific to the S and N antigens. The results are in agreement with NAAT results with the LOD of 0.55 fg/mL for S-protein and 0.016 fg/mL for N-protein, respectively [92]. Table 1 summarize methods applied for the diagnosis of COVID-19 using SARS-CoV-2 antigen proteins. 

Nanomaterial-integrated bioreceptors facilitate the interaction of analytes due to their nanosized nature and high surface-to-volume ratio. The unique physicochemical, optical, mechanical, and magnetic properties of the nanomaterials enable the development of COVID-19 virus detection with improved performances [93]. There are several biosensors developed for the detection of the SARS-CoV-2 for the point-of-care application [94,95]. Nanomaterials enhance the sensitivity and rapid detection of SARS-CoV-2 biosensors. A simple in-house build biosensor device (eConSens) has been developed using Fluorine Doped Tin Oxide (FTO) coupled with gold nanoparticles and nCOVID-19 antibody by Mahari and coworkers. This device specifically detects the nCOVID-19 spike antigen (nCOVID-19 Ag) with high sensitivity with a detection limit of 10 fM in standard buffer and 90 fM in spiked saliva samples within 30 s [96]. Recently, Yang et al. reported AEC-2 functionalized silver nanotriangle (AgNT) array localized surface plasmon resonance (LSPR) sensor for SARS-CoV-2 detection. Spike RBD protein and CoV NL63 proteins were tested and detected with a limit of detection (LOD) of 391 PFU/mL and 625 PFU/mL, respectively, with a detection time of less than 20 min [97].

### 4.3. Biosensors for the Detection of Antibodies

Antibodies produced in response to viral infections can be utilized for diagnosing COVID-19. Viral protein load may change in the due course of time, which makes it difficult to detect SARS-CoV-2 by nucleic acid-based methods, especially in the early and later stages of infection. A high viral load is present in the first week after the symptoms, which starts diminishing later over time [98]. However, antibodies developed in response to the viral proteins would allow a larger time window for detecting SARS-CoV-2 infection. Both IgG and IgM antibodies can be detected one week after COVID-19 infection. IgM level decreases within a short time and IgG levels remain the same up to 5 weeks after infection [99]. The sensitivity of virus detection is 57.2% to 87.5% for IgM and 71.4% to 87.5% for IgG [100]. S-protein RBD showed more antigenicity than N-protein, and the sensitivity of RBD IgM, IgG, and IgA are 96.8%, 96.8%, and 98.6%, respectively [87]. The most prominent challenge in this method is the cross-reactivity of SARS-CoV-2 antibodies with antibodies generated against other coronaviruses such as SARS-CoV, as reported by Lv et al. [101], where there is a significant cross-reactivity of SARS-CoV against the SARS-CoV-2 S-protein antibodies. This antibody-based approach can confirm that the patient has been infected by the virus in the past, which enables us to observe the stages of the infection and identify the people who have developed immunity against SARS-CoV-2. The antibody detection uses the host response against the viral infection, specifically, the antibodies produced against the viral proteins (S, N, E, and M proteins) [85].

A portable and low-cost electrochemical immunosensor for the quantitative detection of IgG and IgM antibodies against spike SARS-CoV-2 protein in human serum has been developed. The sensor can detect both antibodies within 13 min, and is highly stable for up to 24 weeks at room temperature [77]. A label-free, paper-based, electro-chemical biosensor for SARS-CoV-2 antibodies detection was developed, in which the S-protein was immobilized on the electrode. The antibodies specific to S-protein can be recognized by the electrochemical signal response. The LOD of this method is 10.1 ng/mL, which is higher than the LOD required for the detection of antigens from nasopharyngeal swab specimens [102]. However, the sensitivity of the method would be improved further by using updated technologies for future applications. Yousefi et al. have developed a reagent-free electrochemical biosensor for the detection of the whole viral particle using the SARS-CoV-2 spike antibody as a bioreceptor, in which the negatively charged DNA is used as a spacer between the electrode and the antibody as well as a redox probe. This sensor can detect the viral particle within 5 min. Moreover, it is highly stable and there is no significant change in performance after 9 months [103]. Point-of-care lateral flow immunoassay (LFIA) has been developed for the simultaneous detection of immunoglobulin M, IgM and IgG antibodies of SARS-CoV-2 from human blood samples within 15 min. In this method, Au nanoparticles were conjugated with SARS-CoV-2 recombinant protein and rabbit IgG and spread on the conjugate pad. IgG, IgM, and the control lines were immobilized by antihuman IgG, antihuman IgM, and antirabbit IgG. A line color change of pink or red in both test and control lines indicates the presence of the respective antibody, with a specificity and sensitivity of 90.63% and 88.66%, respectively [78]. Superparamagnetic nanoparticles (SMNPs) and giant magnetoresistance (GMR)-based LFIA was developed for the simultaneous detection of both anti-SARS-CoV-2 IgM and IgG [104].

LFIA coupled with a portable spectrometer shows enhanced specificity and sensitivity, and quantitatively detected COVID-19 antibodies (IgG and IgM). The quantification is based on optical density rather than visual interpretation. This method could be applied for the detection of trace amounts of antibodies in the early stage of SARS-CoV-2 infection [105]. Bian et al. have developed fiber optic-biolayer interferometry (FO-BLI)-based biosensors for the rapid detection of SARS-CoV-2, both neutralizing antibodies (NAbs) and binding antibodies (BAbs) from human serum. They used 3,3′-diaminobenzidine to improve the sensor signal to achieve sensitive detection of NAbs and Babs (antireceptor binding domain, anti-RBD) and antiextracellular domain of spike protein (anti-S-ECD) with a sensitivity of 10 ng/mL in a plain buffer as well as a 100-fold diluted serum. The detection time for the NAbs and BAbs are 7.5 and 13 min, respectively. This sensor is robust and doesn’t need sensor surface blocking to prevent the nonspecific adsorption of biomolecules from the serum. The main advantage of these dip-in sensors is that they are easy to handle and routine use for the assay [79]. Anti-SARS-CoV-2 antibody levels have been detected using Janus emulsions or Janus particles as biosensors. Janus emulsions are the combination of immiscible hydrocarbon and fluorocarbon oils, and they are conjugated to a secondary antibody of IgG protein and SARS-CoV-2 spike receptor binding domain (RBD), respectively. When these two types of Janus particles are mixed in the presence of anti-SARS-CoV-2 spike IgG antibody, they are agglutinated by spike IgG antibody, which then binds to the secondary antibody and the SARS-CoV-2 spike protein RBD. In another set of studies, fluorescence dye and blocker dye are conjugated in both phases of the Janus particles, respectively. The blocker dye absorbs both excitation light and the emission from the fluorescent dye. Thus, in the absence of a SARS-CoV-2 spike IgG antibody, the excitation and emission light pass through the blocker dye phase; however, in the presence of an antibody, the orientation of the droplets changes due to agglutination around an anti-SARS-CoV-2 spike antibody. Both of these Janus droplet methods are used for the qualitative and quantitative detection of SARS-CoV-2 spike IgG antibodies with LOD of 0.2µg/mL within two hours [81].

An ACE-2-based biosensor has been developed for sensitive detection of neutralizing antibodies at LOD of 100 ng/mL. This method could also be applied to naturally infected patients and vaccinated persons [80]. A new technology, functional SERS encoded nanoparticles (NPs) (or SERS nanotags), was introduced in the LFIA system to replace the AuNPs used as the signal reporter in general. In this technology, dual-layers of Raman molecule (5,5-dithiobis-(2-nitrobenzoic acid) (DTNB) is loaded inside and outside of the Ag-coated SiO_2_ NPs (SiO_2_@Ag NPs) as advanced SERS tags, which enhance the SERS signals, stability, and the sensitivity of the system. The LOD of this method has improved 800 times compared to the standard Au nanoparticle-based LFIA for the detection of IgM and IgG. This makes AnSERS-LFIA is an efficient, rapid, accurate, and sensitive method for the mass screening of SARS-CoV-2 infections [106]. An opto-microfluidic sensing device was designed for the detection of anti-S-protein SARS-CoV-2 antibodies based on the localized surface plasmon resonance (LSPR) principle. This technology is formed by the integration of Au nanospike-coated glass substrate and the microfluidic chip, connected to a reflection probe for sensing the SARS-CoV-2 spike antibody in the diluted plasma (1:1000) within 30 min. The LOD of this technology was found to be 0.08 ng/mL, which is below the clinically recommended value and user-friendly. Therefore, this technology can be used for the point-of-care application [82,97]. A new time-resolved fluorescence immunoassay (TRFIA) was developed which can specifically sense the COVID-19 anti-N-protein and anti-S-protein total antibodies. The recombinant N- and S-proteins were conjugated with Eu^3+^ chelating agent and coated in a 96-well plate. Antibody-containing samples were incubated, and the fluorescence was measured. TRFIA analysis is as good as the nucleic acid assay kit and has much better sensitivity compared to the colloidal gold kit and chemiluminescent kit [107]. Using three different biomarkers (IgG, IgM, and antigen), detection of SARS-CoV-2 on a single platform has been demonstrated using a point-of-care microfluidic device by Lin et al. This is an integrated system of homemade fluorescence detection analyzer, SARS-CoV-2 diagnostic microchips, and multiple immunoassays. As this device detects three different targets simultaneously, it is more accurate, and specific and can detect all of the biomarkers within 15 min [108]. A new multiplex, xMAP INTELLIFLEX DR-SE flow analyzer, used for the simultaneous detection of specific antibodies developed against SARS-CoV-2 spike (S), receptor binding domain (RBD), and nucleocapsid (N) proteins, was performed using a fluorescent microsphere immunoassay. A highly sensitive surface-enhanced Raman-scattering-based lateral flow immunoassay (SERS-LFIA) has been developed for the detection of anti-SARS-CoV-2 IgM/IgG in parallel. Another interesting multiplex screening test for COVID-19 has been published recently for the quantitative detection of 10 different biomarkers (six viral nucleic acid genes, two spike protein subunits, and two antibodies). This label-free nanoplasmonic biosensor can detect the target at as low as the aM range. The high throughput screening method can quantitatively detect IgM and IgG antibodies from the SARS-CoV-2-positive patient’s plasma sample with more than 96% sensitivity and specificity [109]. A graphene field-effect transistor (g-FET)-based biosensor for the sensitive detection of SARS-CoV-2 antibody was designed in which the g-FET is immobilized with spike S1 protein. When a specific anti-spike-S1 antibody has been recognized, the change in the current was converted into quantifying the antibody present with the LOD of 150 antibodies/100µL. Clinical serum samples can be analyzed using this biosensor within 2 min [110]. Antibody-based technologies used for the detection of SARS-CoV-2 have been highlighted in Table 1.

## 5. Currently Applied Biomarker Detection Methods in COVID-19 Diagnosis

The most common method used for the detection of SARS-CoV-2 is RT-PCR. With high sensitivity and accuracy, many commercial RT-PCR kits have been developed [111]. Though this method is more reliable, the need for well-trained technicians, long analysis time, and high-cost instruments and reagents create limitations that motivate the researchers to search for alternate methods. Next-generation genome sequencing (NGS), a high-throughput technology, is frequently used for the detection of SARS-CoV-2. Tens of thousands of samples can be analyzed in a single run with high accuracy and reliability compared to RT-PCR [112]. However, it has a long analysis period (~2 days) and an expensive experimental setup. CRISPR-based genome detection is one of the most accurate and outstanding technologies for the diagnosis of the SARS-CoV-2 gene. The CRISPR enzyme cuts a part of the gene and produces either fluorescence or dark signals. The SHERLOCK CRISPR SARS-CoV-2 kit has been introduced by USFDA recently to detect a part of (ORF1ab, “O”) gene and the Nucleocapsid (“N”) gene of SARS-CoV-2 (https://www.fda.gov/media/137746/download (accessed 28 July 2022). CRISPR-Cas12-based DETECTR techniques were applied for the detection of SARS-CoV-2 from respiratory swab specimens with an LOD of 10 copies/μL [113]. In another report, the CRISPR-Cas12a-based fluorescence biosensors can detect as low as two copies per sample collected by nasal swab [114]. Loop-Mediated Isothermal Amplification (LAMP) is a widely used technique for SARS-CoV-2 detection which can be an alternative to the PCR method. Though this is not a very accurate method, it can be applied to semiquantitative and qualitative analysis. An improved performance was observed in the reverse transcription loop-mediated isothermal amplification (RT-LAMP) method. With less operation time, it can work at constant temperature, and the results can be observed by the naked eye as a color change. The sensitivity of this method is in the range of a few hundred copies [115,116]. There are many commercially available, government-approved SARS-CoV-2 diagnostic kits based on potential diagnostic biomarkers such as viral RNA, proteins, and antibodies that have been elaborately reviewed recently [34].

## 6. Other Biomarkers for COVID-19 Diagnosis

In addition to viral gene, viral proteins, and antibodies developed against SARS-CoV-2 infection, there are a variety of clinically important biomarkers for the diagnosis of COVID-19, such as reactive oxygen species (ROS) cytokines, C-reactive protein, serum amyloid A, Lactate dehydrogenase, D-dimer, brain natriuretic peptide (BNP) neuron-specific enolase (NSE), and others [117,118]. The concentration level of different inflammatory biomarkers for normal patients and infected patients were compared [119]. Yet, the most accurate and specific biomarkers must be studied critically for clinical application. Smell dysfunction is one of the potential biomarkers for the diagnosis of COVID-19. It is reported that 98% of the patients experience smell dysfunction (58% were anosmic or severely microsmic, 27% with moderate microsmia, 13% with mild microsmia, and 2% normismia) [120]. Quantitative smell tests indicate smell dysfunction, which can diagnose COVID-19 at an early stage and prevent further propagation. The neutrophil-to-lymphocyte (NRL) ratio is also used as a biomarker for poor prognosis and is a potential predictor of the severity and mortality in patients diagnosed with COVID-19 [121]. An elevated level of cardiac troponin is correlated with a poor prognosis. However, further studies have to be conducted for accurate results [122]. D-dimer has been identified as the first accurate biomarker for altered coagulation in COVID-19 and prediction of mortality on admission. The d-dimer value for a healthy person is 0–243 μg/mL, and 1.5 μg/mL is the optimal cutoff value for mortality on admission [123]. Seriously ill COVID-19 patients showed a significantly low level of vitamin A compared to mildly ill ones. In total, 0.2 mg/mL of vitamin A indicates the development of ARDS and high mortality [124]. Fluorescence resonance energy transfer (FRET) assay was used for quantitative detection of SARS-CoV-2 using a total extracellular protease’s proteolytic activity on a specific fluorogenic peptide identified from the library of 115 peptides. The SARS-CoV-2 protease-specific dipeptide is labeled with fluorescence donor, FITC, acceptor, and DABCYL at both ends, and the FITC fluorescence is quenched by FRET. In the presence of the SARS-CoV-2 protease, and the peptide cleaved into two fragments leads to change in the fluorescence signal. The increase in fluorescence is directly proportional to the protease activity. This method can detect 0.9 CFU/mL with minimal cross-reactivity with other coronaviruses [125].

## 7. Artificial Intelligence (AI) and Internet of Things (IoT) in COVID-19 Detection

Modern technologies such as medical image processing, disease tracking computational biology, and medicines and prediction outcomes are used to control/monitor the COVID-19 spread. AI is used to predict future outbreaks and diagnose infections. Drones and robots are used to supply food and medicine and sterilize infected public places [126]. A novel method of COVID-19 detection combines two emerging technologies, nanopores and artificial intelligence, in a platform termed AI-Nanopore. Nanopores are essentially pores of nanometers in size held in materials such as silicon. These pores can function as single-molecule detectors when current is passed through the membrane. Artificial intelligence, on the other hand, is the imitation of human intelligence by computers or machines. AI combines computer science and datasets that the machine trains to “learn” patterns and engage in problem-solving. Extending the science of artificial intelligence beyond what it is traditionally used for, the AI–nanopore platform developed by Taniguchi et al. is a relatively simple platform that eliminates the need for RNA extraction. The platform consists of machine learning (ML) software that sits on a server, a portable high-speed and high-precision current measuring instrument, and scalable, cost-effective semiconducting nanopore modules. The AI–nanopores are successful in accurately detecting four types of coronaviruses (HCoV-229E, SARS-CoV, MERS-CoV, and SARS-CoV-2) of comparable size with a sensitivity of 90% and specificity of 96% in a 5-min assay. Since the AI–nanopore platform relies on data that is fed by changing the training data from cultured viruses to PCR-positive/negative specimens, the AI–nanopore platform can be used to detect both positive and negative specimens again with high sensitivity at high throughput [127].

Fortunati et al. developed another unique setup that involves Wi-Fi (wireless internet connection) enabled IoT (Internet of Things) in a smart and portable electrochemical immunosensor for the quantification of SARS-CoV-2 spike protein with combined ML capabilities. The sensor is based on the immobilization of monoclonal antibodies directed at the SARS-CoV-2 S1 subunit on screen-printed electrodes (SPE) functionalized with gold nanoparticles. The setup combines the ease of using an LFIA strip test with the unique benefits of electrochemical sensors, such as specificity, sensitivity, and accuracy along with obtaining a quantitative response not traditionally offered by the LFIA strip tests. The protocol involves a single-step, one-hour sample incubation on the SPE surface. ML was used to process data, run analyses, classify samples as positive or negative, and improve the accuracy of the measurement. This feature makes the setup comparable to traditional methods (such as RT-PCR) in POC contexts. A dataset of 55 positive and 53 negative samples was used for training and validation purposes. Different SVM classifiers were evaluated in order to select the best SVM kernel and optimal hyperparameters that achieve the highest classification accuracy computed. The test accuracy in terms of true positive/true negative sample classification using the best classification model was about 97.3%. Furthermore, the ML algorithm was integrated into cloud-based portable WiFi devices, which makes the setup smart and portable [128].

## 8. Challenges and Future Prospective

The early-stage detection of SARS-CoV-2 viral infection would prevent further spreading in a community. The most perilous thing about SARS-CoV-2 is the possibility of being asymptomatic, which is an obstacle to monitoring the spread of infection and may lead to increased fatality. The low accuracy, presence of complications in the sample preparation and data analysis, and long processing time are major disadvantages of the current conventional methods. Therefore, it is important to find rapid, low-cost, and mass diagnostic methods to control transmission, by single-step detection, without any pre-sample preparation (RNA extraction) steps and additional signal-enhancing agents. The different diagnostic methods are summarized in Table 2. Rapid biosensing technology plays a crucial role in minimizing respiratory viral disease transmission. Most common SARS-CoV-2 biosensors are designed based on viral components such as viral RNA, N-protein, E-protein, S-protein, M-proteins, and antibodies (IgM and IgG) [129]. 

Among all methods, the nucleic acid-based detection technique is the gold standard for COVID-19 diagnosis. However, this method demands the most intensive laboratory resources, high cost, and long processing time and does not fulfill the demand for a huge number of samples analyzed in a short time. Compared to nucleic acid-based diagnosis, viral protein detection and serology tests are suitable in terms of cost, labor, and assay time. Although these methods meet ASSURED and POC tests, low sensitivity and cross-reactivity are the major limitations. The major challenges in the antibody-based immunosenors are the cross-reactivity of the antibodies raised against closely associated antigens such as SARS-CoV-2 and SARS-CoV. Lv et al. studied antibody responses in 15 serum samples of patients infected with SARS-CoV-2 and 7 infected with SARS-CoV, and showed frequent cross-reactivity of SARS-CoV S-protein antibody with SARS-CoV-2 S-protein antibody to S-protein [101]. Lack of specificity leads to serious issues by false positive and/or false negative results. The immune response by SARS-CoV-2 can be detected within a week or later. The time to develop detectable antibodies and viral loads is another concern for antibody-based detection methods. Moreover, asymptomatic patients infect the neighborhood environment before they are diagnosed [130]. Several optical biosensors (Surface plasmon resonance, fluorescence, colorimetric) have been reported for the detection of SARS-CoV-2. However, designing the sensors in the portable form for the point-of-care application is still challenging. On the other hand, miniaturized electrochemical biosensors are performing well in terms of sensitivity, accuracy, and selectivity [131]. Development of wireless micro-/nanoelectrochemical biosensors would be an ideal solution for detection COVID-19 infection. However, the exclusion of nonspecific adsorption of biomolecules on the electrode surface is a big task in electrochemical biosensors. Another promising approach is to develop a miniaturized sensing platform integrated with a PCR-based amplification system. In this setup, the reagents, programed microfluids, and portable detecting instrument are equipped and the assay results will be reported in a short time [113]. Advancement in the POCT biosensors with multiplexing and high throughput screening can by-pass the time frame for the sample processing time. Integration of both nucleic acid and IgG/IgM antibody tests would be more reliable and accurate to know the early and later stage of COVID-19 infections. Additionally, several other human host biomarkers have been reported, such as neutrophil–lymphocyte ratio (NLR), C-reactive protein (CRP), erythrocyte sedimentation rate (ESR), procalcitonin (PCT), interleukin (IL)-6, D-dimer, troponin, creatine kinase (CK), aspartate aminotransferase (AST), and disseminated intravascular coagulation (DIC). Other novel biomarkers can be identified through the accurate analysis of multiple case studies; in particular, homocysteine and angiotensin II could play a significant role as potential biomarkers [132]. In addition to the existing biosensors, designing novel biomarkers-based biosensors would be another possibility for the rapid diagnosis of SARS-CoV-2. Target-specific single-strand oligonucleotides (Aptamer) are alternatives to antibodies and could be an important candidate for the future perspective for COVID-19 diagnosis. They can be used as a recognition receptor for the detection of SARS-CoV-2 and can efficiently block viral infection [133,134]. Biodegradable porous microneedles might be one of the options for rapid detection of SARS-CoV-2 IgM/IgG in dermal interstitial fluid (ISF) with minimal invasiveness. The unique characteristics of gold and carbon nanomaterials would be integrated for the enhancement of the biosensor’s performance [135]. Though nanomaterial-integrated biosensors have been reported, more work needs to be conducted in order to improve the accuracy and reduce false positives. Several biosensors for COVID-19 detection have been reported, and a limited number of biosensors are available in the market. Optimization of sample preparation, experimental and storage conditions, assay validity, and output of results is still taking place, and this poses a major obstacle for commercialization. Biosensors with a long processing time can be used at POCT, depending upon the demand and the available resources. The obstacles would be overcome by the continuous effort of researchers to create and optimize ideal biosensors for COVID-19 diagnosis. Environmental concern is another factor to be considered. Most biosensors are made of biodegradable and environmentally friendly material. As for the nondegradable biosensors, they can be recycled to minimize the negative impact on the environment.

## Figures and Tables

**Table 1:** Various Biosensors developed for SARS-CoV-2 diagnosis.

SARS-CoV-2 Target	Detection Method	Readout	LOD	Assay Time	Sensitivity and Specificity	Reference
Nucleic acid-based biosensors						
SARS-CoV-2 RNA	DNA caped Au Nanoparticles	Visual	0.18 ng/L	10	-	[37]
Rdrp1, rdrp2, E, N (n1, n2 and n3) genes	RT-PCR	Fluorescence	-	<90 min	94% 100%	[36]
SRAS-CoV-2 RNA (N-Gene)	Swab-to RT-LAMP	Visual	-	-	97.5% 99.7%	[39]
RdRp gene	RT-LAMP	Visual	-	30 min	95.74% 99.95%	[40]
SEAS-CoV-2 RNA	Microfluidic-integrated lateral flow recombinase polymerase amplification (MI-IF-RPA)	Visual	1 copy/µL	30 min	97% 100%	[45]
SARS-CoV-2 RNA	Microfluid integrated LAMP-RPA	Fluorescence	10 copies	60 min	95.83% 94.12%	[46]
SARS-CoV-2 N-Gene	Reverse transcription recombinase-aided amplification coupled with lateral flow dipstick (RT-RAA/LFD)	Visual	1 copy/µL	30 min	100% 100%	[49]
SARS-CoV-2 Orf1ab Gene	Reverse transcription recombinase-aided amplification (RT-RAA)	Fluorescence	0.48/L	25 min	- 100%	[50]
ORF1ab gene	CRISPR/Cas9-mediated triple-line lateral flow assay (TL-LFA) integrated with (RT-RPA)	Visual	100 copies/25 µL	60 min	-	[60]
Viral protein-based biosensors						
S-protein antigen	BERA (bioelectric recognition immunoassay)	Electric biosensor Electrical Signal	1 fg/mL	-	-	[71]
S-protein antigen	Electrochemical technique	Electrical Signal	19 ng/mL	-	-	[72]
S-protein antigen	Graphene field effect transistor (GrFET)	Sensitive graphene field effect transistor	0.2 pM	2 min	-	[73]
N-protein antigen	Molecularly imprinted polymers (MIPs)	Electrical Signal	15 fM	-	-	[74]
N-protein antigen	Electrochemical technique	Electrical Signal	8 ng/mL	-	-	[72]
N-protein antigen	Optical technique	Visual	<100 copies/mL	-	-	[75]
N-protein antigen	Electrochemical	Square wave voltammetry	0.4 pg/mL	-		[76]
Antibody-based biosensors						
S-protein antibody	Label-free paper-based electrochemical biosensor	Electrochemical Signal	10.1 ng/mL	13 min	-	[77]
Antibodies	Lateral flow immunoassay (LFIA)	Visual	-	15 min	88.6% 90.63%	[78]
Neutralizing antibodies	Optic-biolayer interferometry	-	10 ng/mL	7.5–13 min	-	[79]
Neutralizing antibodies	100 ng/mL	Visual	100 ng/mL	-	-	[80]
S-protein antibody	Fluorophore conjugated Janus emulsion particle	Optical Image and Fluorescence	200 ng/mL	120 min	-	[81]
S-protein antibody	Opto-microfluidic sensing device	LSPR	0.08 ng/mL	30 min	-	[82]
S-protein antibody	Electrochemical (differential pulse voltammetry)	Electrochemical Signal	0.3 fg/mL	20 min	-	[83]

**Table 2:** Diagnostic approaches to detect COVID-19.

Biological Component	Diagnostic Approach	Method of Detection	Reaction Time	Advantages	Disadvantages
Imaging for medical diagnosis	X-ray/CT scan	Chest	1 h	More sensitive to the status of the disease’s infection and organ damage. Combined with RT-PCR enhance the sensitivity	Unable to differentiate between different viral-mediated pneumonia. Equipment is expensive, needs well-trained expert to operate
Serological parameters	Rapid antibody test (IgG and IgM)	IgG and IgM levels in serum	20–30 min	Rapid, identification of specific viral infection	Less sensitivity and specificity. False positive. Unstable, not suitable to storage for long time
Viral genome	Nucleic acid Amplification/Sequencing	RNA amplification (RT-PCR)Genome sequencing (NGS) RT-LAMP, CRISPER	5–6 h ~2 days 1–2 hrs	Gold standard for viral detection, High selectivity and specificity. High accuracy High selectivity and specificity	Expensive, laborious, time-consuming, needs trained technicians. Unable to detect postinfection stage Probability for contamination, false positive. Primer design is complicated
Viral proteins	Viral (S, N, E, and M) Proteins	Lateral flow immunoassay	20–30 min	Fast and low cost, no need of sample pretreatment, moderate specificity and sensitivity, easy to execute. Immunity against the infection	No information about the early infection stage. Long time storage at room temperature is not possible. Possibility of false positive
		Optical, electrochemical, and microfluidic biosensors	2–20 min	Fast and low cost, no need for sample pretreatment, multiple sample analysis, high specificity and sensitivity, easy to execute. Can be integrated with any platform	Needs more attention to obtain accurate results. Adsorption of nonspecific molecules on the electrode. Autofluorescence from nonspecific biomolecules. Miniaturizing, scaling up, and commercialization is challenging

## Data Availability

Not applicable.
